# Dynamic Lifestyle Changes Mitigate Alzheimer's Disease Risk Across Genetic Susceptibility

**DOI:** 10.1002/alz70860_097792

**Published:** 2025-12-23

**Authors:** Yue Shi, Takuji Kawamura, Yingxu Liu, Kentaro Oba, Yasuyuki Taki

**Affiliations:** ^1^ Tohoku University, Sendai, Miyagi, Japan; ^2^ Smart‐Aging International Research Center, Tohoku University, Sendai, Miyagi, Japan; ^3^ Mark and Mary Stevens Neuroimaging and Informatics Institute, University of Southern California, Los Angeles, CA, USA

## Abstract

**Background:**

Genetic predisposition is a well‐established risk factor for Alzheimer's disease (AD), with polygenic risk scores (PRS) providing a quantitative measure of genetic susceptibility. Lifestyle factors, including smoking, alcohol consumption, physical activity, and body mass index (BMI), also contribute to AD risk. However, the interplay between genetic risk and lifestyle changes, as well as their combined effect on AD risk and cognitive function, remains unclear. This study incorporates epigenetic age acceleration (EAA), a marker of biological aging, to explore how longitudinal changes in lifestyle factors influence AD outcomes and cognitive decline.

**Method:**

We analyzed data from participants in the Health and Retirement Study (HRS), including diverse racial and ethnic groups. The analysis is based on data from wave 13 (2016), with a sample of *N* = 7,265 participants and an average age of 73.2 years (age range: 28‐111 years). We derived lifestyle risks, including physical activity, social activity, and smoking, from longitudinal data collected since wave 7 (2004). AD diagnosis was assessed longitudinally based on self‐reported measures. Cognitive function was assessed using validated telephone‐based cognitive tests from 2016 to 2020. EAA was quantified using DNA methylation clocks, specifically derived from GrimAge and DunedinPoAm38 residuals, to capture biological aging.

**Result:**

Lifestyle improvements, particularly increased physical activity, smoking cessation, and moderate alcohol consumption were associated with slower cognitive decline and reduced AD risk across all genetic risk groups. PRS strongly predicted worse cognitive outcomes and higher AD risk. Notably, the interaction between physical activity and PRS indicated that higher physical activity may buffer the negative impact of genetic susceptibility on cognitive function. Among individuals with high genetic risk, positive lifestyle changes were linked to better language and executive function scores.

**Conclusion:**

This study integrated EAA with longitudinal analyses of lifestyle and genetic factors, providing novel insights into the pathways connecting lifestyle changes, genetic susceptibility, and cognition. Our findings underscore the importance of targeted lifestyle interventions in mitigating AD risk even among individuals with high genetic risk, highlight the potential for personalized prevention strategies based on biological aging and genetic profiles.

